# P-730. Recurrent Gonorrhea and Chlamydia Infections in Cisgender Women: Opportunities for Prevention and Intervention

**DOI:** 10.1093/ofid/ofaf695.941

**Published:** 2026-01-11

**Authors:** Kastin Pan, Sarah Naz-McLean, Alexis Rooney, Ann E Woolley, Lisa A Cosimi

**Affiliations:** Brigham and Women's Hospital, Boston, MA; Brigham and Women's Hospital, Boston, MA; Brigham and Women's Hospital, Boston, MA; Brigham and Women's Hospital, Boston, MA; Brigham & Women's Hospital, Boston, Massachusetts

## Abstract

**Background:**

Effective prevention interventions are critical to reducing the burden of sexually transmitted infections (STIs) including gonorrhea (GC) and chlamydia (CT). Recent advances have focused on strategies such as post-exposure prophylaxis. However, recommendations vary by population and remain understudied in cisgender women. Identifying risk factors for recurrent GC and CT infections in women is critical to guiding targeted prevention interventions and evaluating determinants of uptake and effectiveness in diverse populations.

**Figure:**
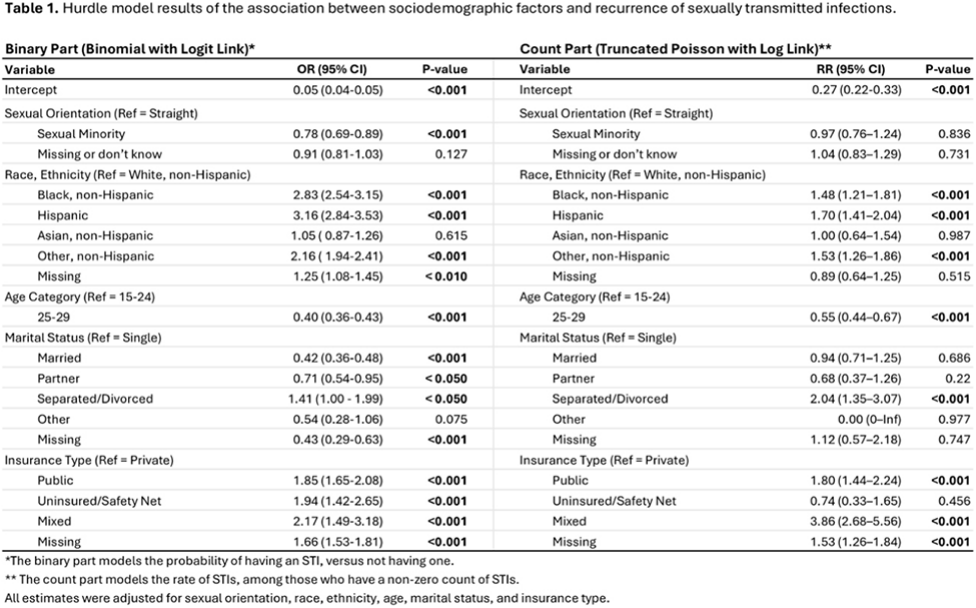

**Methods:**

Data from the Mass General Brigham integrated healthcare system included cisgender women aged 15 to 29 who tested for GC/CT from January 2016 to January 2024. Laboratory-confirmed infections were counted per unique patient, excluding tests within 30 days of a diagnosed infection, which were considered continuing cases. Using a hurdle model to account for excess zeros (i.e., non-infections), we evaluated the association between sociodemographic factors and number of STIs. The model’s two-part structure included: 1) the binary odds of any infection vs. none, and 2) the infection counts among women with ≥1 diagnosis.

**Results:**

Of 56,966 women tested, 3,374 (5.9%) had a positive result – 362 (0.64%) for GC and 3,184 (5.6%) for CT. Among those infected, 616 (18.3%) were reinfected during the study period.

Among women with at least one infection, the risk of recurrent infection was 70% higher in Hispanic women (RR = 1.70, 95% CI: 1.41–2.04) and 48% higher in Black women (RR = 1.48, 95% CI: 1.21–1.81) than non-Hispanic White women. Women aged 25–29 had a 45% lower risk than those aged 15 – 24 (RR = 0.55, 95% CI: 0.44–0.67).

Separated or divorced women had twice the risk of reinfection compared to single women (RR = 2.04, 95% CI: 1.35–3.07). Compared to women with private insurance, those with mixed insurance had a fourfold higher risk (RR = 3.86, 95% CI: 2.68–5.56), while those with public insurance had nearly double (RR = 1.80, 95% CI: 1.44–2.24).

**Conclusion:**

Disparities in recurrent STIs associated with race and ethnicity, age, marital status, and insurance type highlight the need to prioritize cisgender women at increased risk in future targeted prevention and intervention research.

**Disclosures:**

All Authors: No reported disclosures

